# Compulsory Vaccination: The Limit between Public and Private

**DOI:** 10.1055/s-0040-1722214

**Published:** 2020-12-21

**Authors:** Cecilia Maria Roteli-Martins, Júlio César Teixeira

**Affiliations:** 1 Faculdade de Medicina do ABC, Santo André, SP, Brazil; 2 Universidade Estadual de Campinas, Campinas, SP, Brazil

The first vaccine was created by the British physician Edward Jenner in 1796 to prevent smallpox, demonstrating that inoculating material from a lesion could protect against a subsequent infection, thereby beginning a new era. A century later, new vaccines were developed with a relevant impact on the occurrence of a large number of infectious diseases, many of them eradicated, and the collective effect of achieving high vaccine coverage became evident. The United States Center for Disease Control (CDC) declared that vaccines represent one of the ten most valuable purchases of the 20 ^th^ century, compared to drinking water. 1

Brazil has one of the most complete population vaccination programs and, mainly, accessible. In recent years, the refusal to receive vaccination and the consequent decline in herd or population immunity have contributed to the return of infectious diseases already under control, through numerous outbreaks with harm to public health, causing polarized debates among groups in favor and against vaccines. 2 In the meantime, in order to protect the individual and the population and justify the continuation of the current vaccination program, the Brazilian Justice has been called several times. Currently, there is jurisprudence for the compulsory vaccination of children according to the vaccination schedule of the National Vaccination Program of the Ministry of Health. 3

Nevertheless, with the globalization of access to information over the internet, there is a mismatch between knowledge and emotional vulnerability leading to the emergence of opportunists who make use of “fake news” to reverberate old and already defined situations that seem new for many people, leading to the start of some debates again. The current scientific, political and ethical challenges faced in dealing with refusal to vaccinate have been reported in previous decades. The issue raised is the balance of compulsory and coercive vaccination. Is it reasonable to impose compulsory vaccination?

In Brazil, the smallpox vaccination was declared mandatory for children in 1837 and for adults in 1846. However, this resolution was not enforced, also because the vaccine production on an industrial scale in Rio did not start until 1884. In June 1904, Oswaldo Cruz motivated the government to send a project to the Congress in which mandatory vaccination would be established throughout the national territory. Only individuals who proved they were vaccinated would be able to have employment contracts, travel authorization, enroll at school, etc. Even with the growing number of smallpox cases in Rio de Janeiro, part of the population rejected the vaccine, considered to be liquid from the pustules of sick cows, and there was a rumor that people who were vaccinated would develop bovine features. 4

Then, the Anti-Vaccination League was created. It united the political agitation and the vaccine refusal in an episode known as the “Vaccine Uprising”, in which several conflicts occurred, with the struggle between military and insurgent forces. After a total balance of 945 people arrested, 461 deported, 110 injured and 30 killed in less than two weeks of conflict, President Rodrigues Alves was forced to give up mandatory vaccination. Later, in 1908, when the city was hit by the most violent smallpox epidemic in its history, people rushed to be vaccinated, in a contrary direction to the episode of the Vaccine Uprising. 4

The effectiveness of the measure was demonstrated with the disease eradication, showing that high vaccination rates lead to the protection of the entire community.

In December 2019, we began to hear about a new virus that presented itself as highly infectious, the SARS-CoV-2. In the following months, every day the scientific community woke up with new data on morbidity and mortality. From astonishment to the declaration of a pandemic by the World Health Organization (WHO), all researchers and large laboratories started looking for treatments and vaccines to fight the new disease.

In our current scenario, COVID-19, caused by the infection of the new coronavirus, is spreading on all continents and so far, there is still no vaccine with proven efficacy and safety to fight it, despite the advanced stage of clinical research.

With the promise that some formulations will be available in the first half of the year 2021, some questions emerge and touch the role of mandatory vaccination to protect the community, with the social interest conflicting with the individual interest.

Is it desirable to have a community free from an infectious and deadly disease as a result of a high vaccine coverage? The answer is “yes”, but again, the so-called anti-vaccine groups are beginning to move to disseminate fanciful versions through social networks, anticipating the debate between mandatory vaccination and the own conviction of each individual. A new plan of action against vaccines is now seeking to manipulate a larger proportion of the population by saying that once vaccinated, we will all be at risk of serious adverse events.

A secondary and also bad effect of the current pandemic was the significant drop in Brazilian vaccine coverage due to restrictive circulation measures that brought concern to health authorities about the upsurge of diseases that were already close to eradication by vaccination.

In view of the above, the two arguments - the obligation and the conviction of being immunized by a vaccine that proves effective and safe against COVID-19 - make this debate important and necessary. The balance between the two can be an important reinforcement to achieve high coverage and consequently, the immunization of the entire community.

A law determining mandatory vaccination, never with violence, might be necessary to guarantee vaccination and a safe social life. However, convincing the population by information and education must always be part of the essential instruments for a better understanding of the positive value of vaccination and thus, maintain the confidence of most Brazilians in vaccines and in the health professionals who recommend them.
